# Genome-Wide Admixture and Association Study of Serum Selenium Deficiency to Identify Genetic Variants Indirectly Linked to Selenium Regulation in Brazilian Adults

**DOI:** 10.3390/nu16111627

**Published:** 2024-05-26

**Authors:** Ligia Moriguchi Watanabe, Lisete Sousa, Francisco M. Couto, Natália Yumi Noronha, Marcela Augusta de Souza Pinhel, Gleyson Francisco da Silva Carvalho, Guilherme da Silva Rodrigues, Carlos Roberto Bueno Júnior, Leslie Domenici Kulikowski, Fernando Barbosa Júnior, Carla Barbosa Nonino

**Affiliations:** 1 Division of Nutrition and Metabolism, Department of Health Sciences, Ribeirão Preto Medical School, University of São Paulo—FMRP/USP, São Paulo 14049-900, Brazil; carla@fmrp.usp.br; 2 Departamento de Estatística e Investigação Operacional (DEIO) e Centro de Estatística e Aplicações (CEAUL), Faculdade de Ciências da Universidade de Lisboa, Campo Grande, 1749-016 Lisbon, Portugal; lmsousa@fc.ul.pt; 3 LASIGE, Departamento de Informática, Faculdade de Ciências da Universidade de Lisboa, Campo Grande, 1749-016 Lisbon, Portugal; fjcouto@ciencias.ulisboa.pt; 4 Department of Internal Medicine, Ribeirão Preto Medical School, University of São Paulo—FMRP/USP, São Paulo 14049-900, Brazil; natty.yumi@gmail.com (N.Y.N.); marcelapinhel@yahoo.com.br (M.A.d.S.P.); 5 Department of Pathology, São Paulo Medical School, University of São Paulo—FMUSP, São Paulo 01246-903, Brazil; gleyson.carvalho@usp.br (G.F.d.S.C.); lesliekulik@gmail.com (L.D.K.); 6 School of Physical Education and Sport of Ribeirão Preto, University of São Paulo, São Paulo 14040-900, Brazil; guirodrigues@usp.br (G.d.S.R.); buenojr@usp.br (C.R.B.J.); 7 Department of Clinical and Toxicological Analyses and Bromatology, Faculty of Pharmaceutical Sciences of Ribeirão Preto, University of São Paulo—FCFRP/USP, São Paulo 14040-900, Brazil; fbarbosa@fcfrp.usp.br

**Keywords:** selenium deficiency, GWAS, admixed population

## Abstract

Blood selenium (Se) concentrations differ substantially by population and could be influenced by genetic variants, increasing Se deficiency-related diseases. We conducted a genome-wide association study (GWAS) to identify single nucleotide polymorphisms (SNPs) associated with serum Se deficiency in 382 adults with admixed ancestry. Genotyping arrays were combined to yield 90,937 SNPs. R packages were applied to quality control and imputation. We also performed the ancestral proportion analysis. The Search Tool for the Retrieval of Interacting Genes was used to interrogate known protein–protein interaction networks (PPIs). Our ancestral proportion analysis estimated 71% of the genome was from Caucasians, 22% was from Africans, and 8% was from East Asians. We identified the SNP rs1561573 in the TraB domain containing 2B (*TRABD2B*), rs425664 in MAF bZIP transcription factor (*MAF*), rs10444656 in spermatogenesis-associated 13 (*SPATA13*), and rs6592284 in heat shock protein nuclear import factor (*HIKESHI*) genes. The PPI analysis showed functional associations of Se deficiency, thyroid hormone metabolism, NRF2-ARE and the Wnt pathway, and heat stress. Our findings show evidence of a genetic association between Se deficiency and metabolic pathways indirectly linked to Se regulation, reinforcing the complex relationship between Se intake and the endogenous factors affecting the Se requirements for optimal health.

## 1. Introduction

Selenium (Se) is an essential nutritional trace element, and its beneficial health effects strongly depend on its concentration [1]. Se is present in nature and organisms in organic and inorganic forms [2]. Due to the distinct metabolic pathways of Se compounds from the diet, the absorption, incorporation into selenoproteins, and urinary excretion may vary, influencing the status of total Se and its metabolites in the body [3]. Therefore, the identification and quantification of Se are of great importance since the relationship between Se level and health effects, represented by a U-shaped curve, suggests illnesses associated with deficiency and excess [3,4]. Complexities in the relationship between Se and health effects have imposed challenges to achieving the optimal concentration range for the human body that could generate more appropriate dietary recommendations [4,5].

The primary role of Se activity is its presence in the catalytic sites of several selenoproteins [3,4]. In eukaryotic cells, Se can be incorporated into 25 human selenoproteins that play diverse roles in the body, including antioxidant action (glutathione peroxidases, GPX), Se transport and storage (selenoprotein P, SELENOP), redox signaling (thioredoxin reductase, TXNRD), thyroid hormone metabolism (iodothyronine deiodinases, DIO), protein folding (15 kDa selenoprotein, SELENOF), among others [3].

Blood Se concentrations tend to differ substantially by population and mainly depend on recent dietary intake and the Se chemical form [4,5]. In the Brazilian population, deficiency is more commonly found, possibly due to food sources from predominant Se-deficient soils in top agricultural regions and socio-economical patterns affecting food choices [6]. However, endogenous factors, such as health conditions, personal characteristics, and genetic variations, may also influence its status in the organism [7,8]. Notably, studies have focused on identifying genetic variations that impact endogenous factors affecting circulating Se concentrations and selenoproteins’ structure, function, and synthesis [7,9,10,11].

Over the past decade, genome-wide association studies (GWASs) have successfully identified associations between genetic variants, human complex diseases, and phenotypic traits [12,13]. Previous GWASs of blood or toenail Se levels in response to Se supplementation were conducted, but mostly in European descent and Se-sufficient populations [7,9,11,14]. However, the GWAS of Se deficiency in an admixed population has not yet been investigated.

Admixed populations are a peculiar case for genetic ancestry inference. The genomes of admixed individuals are a redistribution of genetic variation observed in parental populations, which produces new genomic combinations of pre-existing genetic variants [15]. Thus, genetic ancestry inference in an admixed population is an essential tool to control the effect of population stratification in association studies, identify disease-associated genes for precision medical care, and reveal population history [15]. Brazilians generally trace their origins to the original Amerindians and two primary sources of immigration: Africans and Europeans [16]. More recently, it has also received contributions from other regions, including East Asia and the Middle East [15].

Understanding how different marker sets are assigned to Brazilian genetic ancestry is highly relevant to exploring complex traits in this population [9,15,16]. Also, considering the higher prevalence of Se deficiency in the Brazilian population [6] and the consequences to health [4], identifying genetic variants associated with disorders in Se homeostasis could help to identify individuals with a risk of deficiency. Thus, we conducted a GWAS to identify genetic variants associated with serum Se deficiency in 382 Brazilian descendants.

## 2. Materials and Methods

### 2.1. Ethic Statement

All procedures in this study follow the ethical standards in the Declaration of Helsinki. The study protocol was approved by the Ribeirão Preto Medical School Ethics Committee of the University of São Paulo, Brazil (protocol number CAAE: 14275319.7.0000.5440). Informed consent was obtained from all individual participants.

### 2.2. Study Population

**Figure 1:**
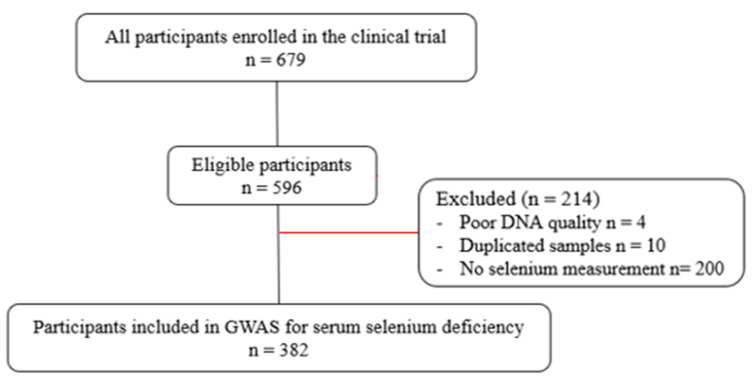
Flowchart of participants’ inclusion.

This observational, cross-sectional study was conducted at the Ribeirão Preto Medical School, University of São Paulo, Brazil. Participants were volunteers from the Metropolitan Region of Ribeirão Preto, Southeastern Brazil. They were adults of both sexes, in all classes of Body Mass Index (BMI), and clinically stable. Patients taking multivitamins and mineral supplements, in use of antibiotics or other medications that are metabolized by cytochrome P4503A4 (CYP3A4), in current tobacco and alcohol consumption, athletes or individuals practicing intense physical activity, or with infectious diseases or with a diagnosis of chronic disease that may interfere with data collection were not included in the study. A total of 679 participants met the inclusion criteria. After removing patients without clinical evaluation, 596 participants were eligible. Participants with poor DNA quality, duplicated samples, and no Se measurement were also excluded, and 382 individuals were considered for analysis in the present study (Figure 1).

### 2.3. Anthropometric Evaluation

A dietitian measured the weight on an electronic platform scale with a precision of 0.1 kg, a maximum capacity of 300 kg, and height with a vertical shaft with 0.5 cm graduation. BMI was calculated by dividing the body mass by the square of the body height, universally expressed in units of kg/m^2^.

### 2.4. Determination of Se Levels

The total concentration of serum Se was determined by an Inductively Coupled Plasma Mass Spectrometry fitted with a dynamic reaction cell (Perkin Elmer Sciex, Norwalk, CT, USA). Samples were diluted in 1:50 with a solution containing Triton X-100 (Sigma-Aldrich, Saint Louis, MO, USA) 0.01% (*v*/*v*), HNO_3_ (Sigma-Aldrich, Saint Louis, MO, USA) 0.05% (*v*/*v*), and 10 mg/L^−1^ rhodium (Sigma-Aldrich, Saint Louis, MO, USA) as an internal standard. The concentration of the analytical calibration standards ranged from 0 to 50 mg/L [17].

### 2.5. Selenium Intake

Se intake was assessed by food record over three non-consecutive days. Patients were oriented to self-record the type and amount of food and beverages consumed. The information was processed in the nutritional analysis program Dietpro version 5i (Dietpro, Viçosa, Brazil).

### 2.6. Exploratory Analysis

The exploratory analysis was performed using the RStudio statistical software (R version 1.1.456). The quantitative variables were expressed as means and standard deviations (SD). Categorical variables were expressed as absolute and relative frequencies.

### 2.7. Genotype Data

Genomic DNA was isolated from whole blood using a PureLink Genomic DNA kit (Invitrogen, Life Technologies Inc., Carlsbad, CA, US), and the concentration was measured using a NanoDrop ND 1000 spectrophotometer (Thermo Fisher Scientific, Waltham, MA USA). According to each protocol guide, genotype data were generated using the Infinium Human Omni5-4 (v1.1 and v1.2) and Infinium Global Screening Array-24 (v3.0 and v2.0) (Illumina, San Diego, CA, USA).

### 2.8. Genotype Data Processing and Association Analysis

After genotyping, data were merged and submitted to the quality control (QC) stage. Samples with more than 10% failure to obtain genotypes were excluded from the sample. In addition, markers with a call rate greater than or equal to 95% and an MAF (minor allele frequency) greater than 1% were considered for analysis only since markers with low frequency or low coverage are not informative. To optimize the results, we performed a k-nearest neighbor (knn) imputation step by identifying and computing the neighboring markers by calculating the Euclidean distance between the markers [18]. All QC and imputation steps were applied through functions implemented in the package ‘snpReady’ version 0.9.6, available in CRAN [19], and ‘impute’ version 1.76, available in Bioconductor [18]. Moreover, to detect genotype calling errors, the Hardy–Weinberg Equilibrium (HWE) was tested for all the bi-allelic SNP markers using the Exact Tests of HWE proposed by Wigginton, Cutler, and Abecasis (2005) [20]. SNPs in the dataset under HWE (*p* ≥ 0.05) were kept from further analyses.

After the QC step, 90,937 SNPs were identified, and we moved forward to the associative analysis using a case-control design. For this step, metadata were created, including sex, age, BMI, and Se levels. To identify markers potentially associated with Se deficiency, we performed an extended version of genome-wide mixed-model association analysis using the following equation:

y=Xβ+Sα+Zu+e

In this equation, ***y*** is a vector of phenotypic observation; ***β*** is a vector of the fixed effect: sex, age, and BMI; ***α*** is a vector of SNP effect; ***u*** is a vector of polygene background effects; ***e*** is a vector of residual effects; and *X*, *S*, and *Z* are incidence matrices of 1s and 0s relating ***y*** to ***β***, ***α***, and ***u***, respectively. The variances of the random effects are assumed to be $Var(u)=2\;K\sigma_{u}^{2}$, and $Var(e)=I\sigma_{e}^{2}$, where *K* is an *n* × *n* matrix of relative kinship coefficients that define the degree of genetic covariance among individuals; *I* is an *n* × *n* matrix in which the off-diagonal elements are 0 and the diagonal elements are the reciprocal of the number of observations for which each phenotypic data point was obtained; $\sigma_{u}^{2}$ is the genetic variance; and $\sigma_{e}^{2}$ is the residual variance. Functions implemented in package ‘SNPassoc 2.0.11’ available on CRAN were used [21,22].

We also calculated the genotypic Odds Ratio (OR) for each SNP genotype and selected the SNPs with OR greater than 1.00 (*p* < 0.05) [22].

The Search Tool for the Retrieval of Interacting Genes (STRING—version 11.5) was used to interrogate known protein–protein interaction networks (PPIs). The STRING database provides critical assessment and integrates all direct and indirect PPIs covering 67,592,464 proteins from 14,094 organisms [23]. An enrichment p-value is calculated by comparing the observed frequency of an annotation term with the frequency expected by chance. A threshold for significant enrichment was set at *p*-value < 0.05 after adjustment with the Benjamini–Hochberg procedure.

### 2.9. Admixture Analysis

We implemented an ancestral proportion analysis method, EIGMIX [24], an accurate and efficient algorithm that can handle millions of variants and many individuals. EIGMIX derives principal components from surrogate populations with reported ancestry and projects an individual of interest to the principal components to determine its origin. Assuming center coordinates of each surrogate population as unit vectors in ancestral proportion space (e.g., (1,0,0), (0,1,0), (0,0,1) for three ancestral populations), EIGMIX builds linear transformation from the principal component space to ancestral proportions, which is used to calculate the proportions of ancestral populations for an individual [24]. All eigenanalysis in the study was in the R package ‘SNPRelate’ [25].

As surrogate populations for EIGMIX, we used the five continental level populations in the 1000 Genomes Project Phase 3 dataset—i.e., African, American, East Asian, European, and South Asian [26]. We processed the SNP array and reference datasets using previous processing recommendations and pipelines [27].

## 3. Results

### 3.1. Population Characteristics and Structure

A total of 382 participants were included and completed all the study analyses. Among them, 62 (16.2%) were men, and 320 (83.8%) were women. The participants’ mean age was 46.9 ± 14.7 years. According to mean BMI, patients were classified as obese (36.1 ± 12.8 kg/m^2^). The mean serum Se concentration (74.1 ± 31.7 μg/L) was lower than the optimal range, especially to reach the saturation of glutathione peroxidase and selenoprotein P (SELENOP), 70–90 μg/L and 100–120 μg/L, respectively [5,28,29]. In association analysis, serum Se levels <70 μg/L were applied as the threshold to assess Se deficiency. Considering this threshold, 185 (48.4%) individuals were classified as Se-deficient. According to the analysis of non-consecutive dietary food records, participants’ Se intake was 62.4 ± 36.4 μg/day, considered adequate according to the Dietary Reference Intake (55 μg/day).

**Figure 2:**
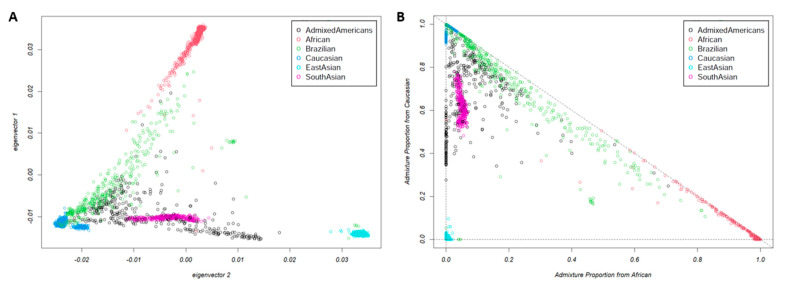
The principal component analysis (PCA) on the Brazilian population and 1000 Genome populations, including Caucasian, African, Admixed American, East Asian, and South Asian, using the complete set of 805,103 SNPs: (**A**) the first and second eigenvectors; (**B**) a linear transformation of coordinate from (**A**) followed by a translation, assuming three ancestral populations with surrogate samples: Caucasian, African, and East Asian. Each point represents one individual.

**Figure 3:**
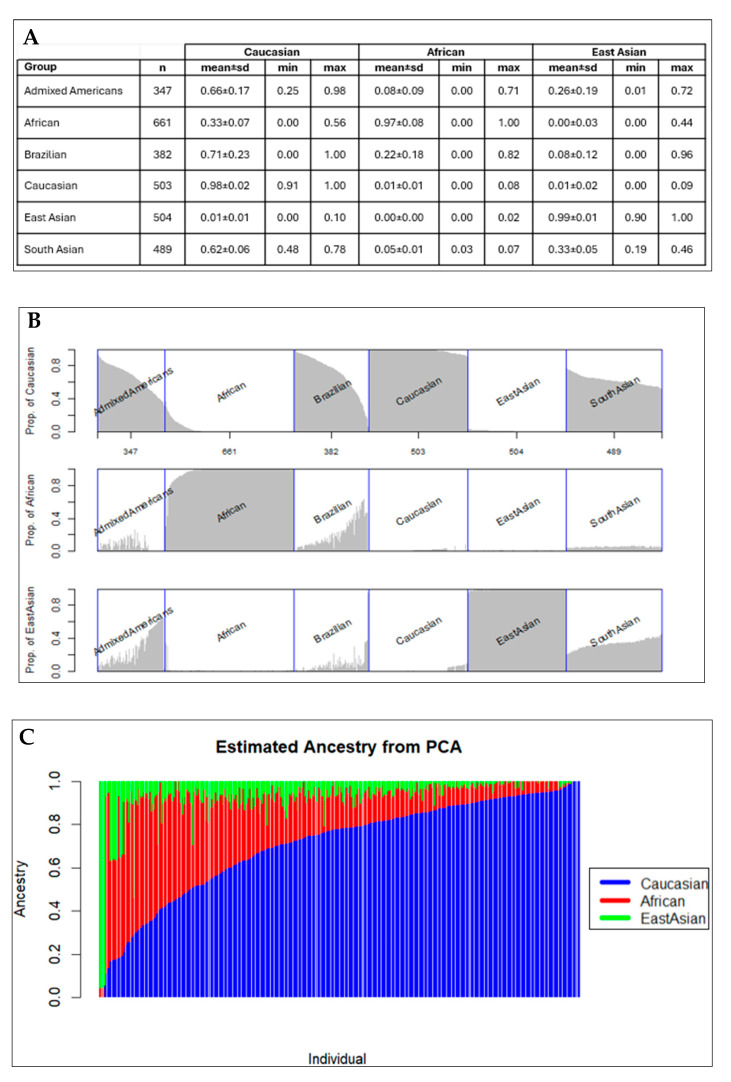
Genotype-based ancestral proportion analysis: (**A**) Mean and standard deviations (sd) of 3 ancestral proportions (Caucasian, African, East Asian) of the Brazilian population and the five continental level populations in the 1000 Genomes Project Phase 3 dataset (Admixed Americans, African, Caucasian, East Asian, and South Asian). (**B**) Graphical representation of the ancestral proportion analysis. (**C**) Estimated ancestry of ancestral proportion from PCA in the Brazilian population, emphasizing the ancestry of Caucasians.

The results from PCA analysis used to detect population structure down to the level of the reference dataset are shown in Figure 2. The ancestral proportion analysis obtained throughout the EIGMIX method is demonstrated in Figure 3. The genotype-based ancestral proportion analysis for admixture populations is estimated by averaging ancestral proportions of individuals using the complete SNP set. Our study confirmed that Brazilians are an admixed population, estimated to have 71% of the genome from Caucasians, 22% from Africans, and 8% from East Asians.

### 3.2. GWAS

**Figure 4:**
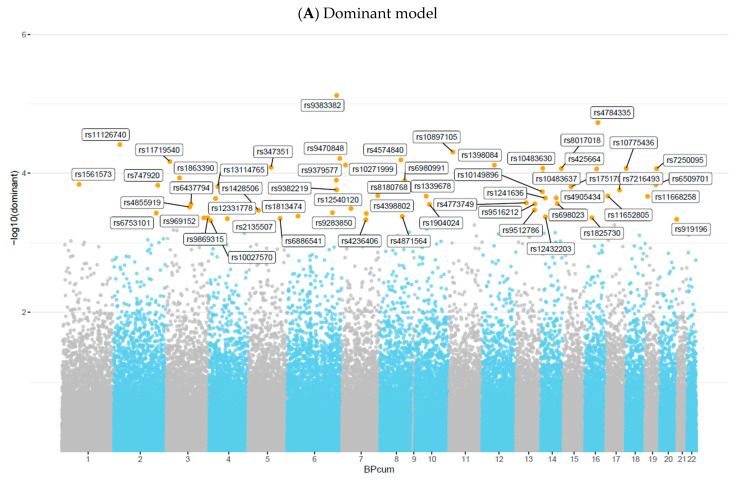

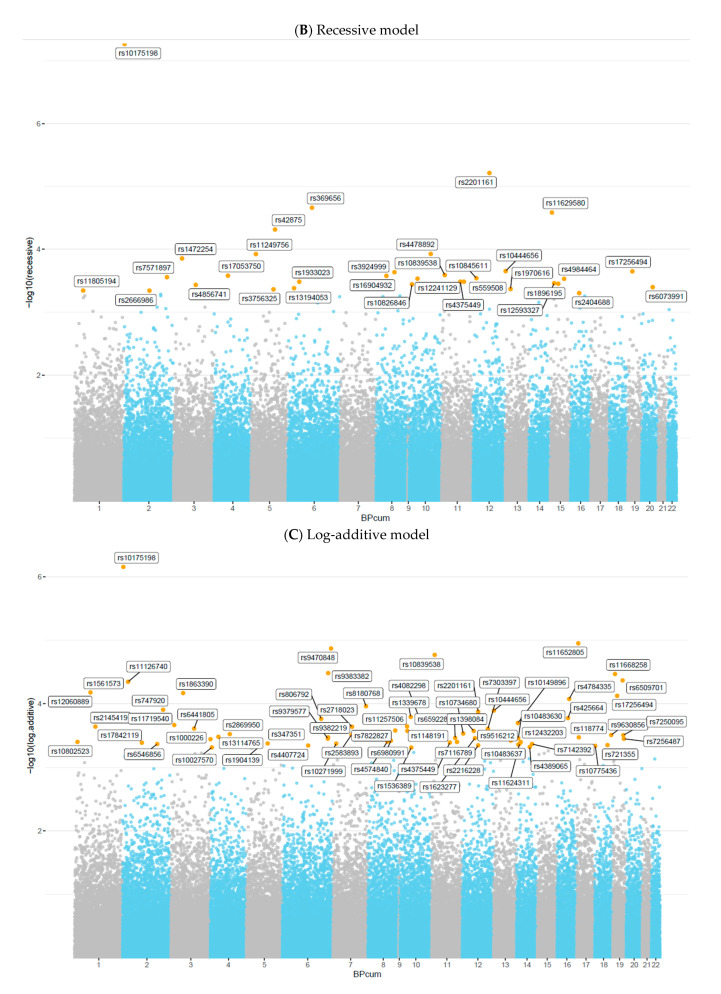
Manhattan plot of 90,937 SNPs showing genome-wide association analysis results for serum selenium concentrations, assuming (**A**) Dominant model, (**B**) Recessive model, or (**C**) Log-additive model, adjusted by BMI, sex, and age. The *x*-axis represents chromosomal positions, and the *y*-axis shows *p*-values on a logarithmic scale.

In the present genome-wide mixed-model association analysis for serum Se deficiency, no association met GWAS at a significance level of 5.0 × 10^−8^. Nevertheless, based on previous studies, we considered a 5.0 × 10^−4^ threshold as a suggestive significance level to test for genetic overlap between SNPs and Se deficiency [7,9,11,30,31,32]. Overall, mixed-model association analysis identified 57 SNPs in the dominant model (Figure 4A), 32 SNPs in the recessive model (Figure 4B), and 67 SNPs in the log-additive model (Figure 4C). The maximum-statistical analysis also identified 162 SNPs that reached a *p*-value < 5.0 × 10^−4^, among which 44 SNPs were in common with those found in mixed-model association analysis (Supplementary Table S1) after Odds Ratio (OR) calculation (Supplementary Tables S2–S4), and 12 SNPs had ORs greater than 1.00 and were selected for further analysis (Table 1).

Although our GWAS analysis identified 12 SNPs associated with Se deficiency, the post-GWAS analysis only found functional biological associations with 4 SNPs: rs1561573 in the gene TraB domain containing the 2B (*TRABD2B*), rs425664 in the gene MAF bZIP transcription factor (*MAF*), rs10444656 in the gene spermatogenesis-associated 13 (*SPATA13*), and rs6592284 in the gene heat shock protein nuclear import factor (*HIKESHI*), described in detail below. We did not identify any biological association between genes H3 clustered histone 4 (*H3C4*), orofacial cleft 1 candidate 1 (*OFCC1*), protocadherin related 15 (*PCDH15*), ankyrin repeat and sterile alpha motif domain containing 1B (*ANKS1B*), syntaxin binding protein 6 (*STXBP6*), sterile alpha motif domain containing 4A (*SAMD4A*), and neurexin 3 (*NRXN3*), and Se metabolism (*p* > 0.05). Furthermore, the SNP in Zinc Finger Protein 320 (*ZNF320*) is a synonymous variation in which the codon substitutions do not change the encoded amino acid, with no effect on the properties of the synthesized protein.

**Figure 5:**
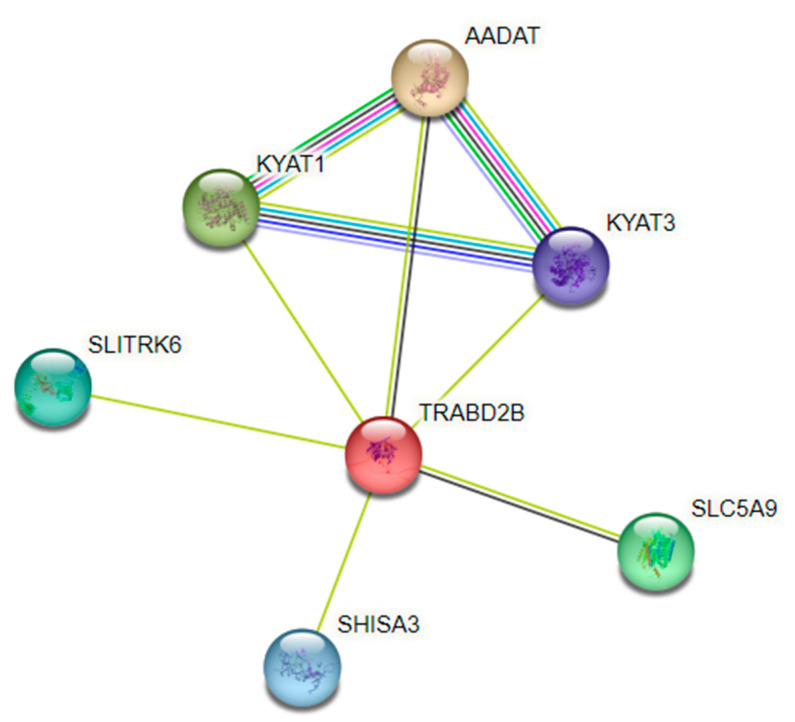
*TRABD2B*, *AADAT*, *KYAT1*, and *KYAT3* genes in a protein–protein association network, using String (PPI enrichment *p*-value: 2.82 × 10^−5^). Network nodes represent proteins. Node Color: colored nodes represent query proteins and the first shell of interactors. Edges represent protein–protein associations. Known Interactions—light-blue line: from curated databases; pink line: experimentally determined. Predicted Interactions—green line: gene neighborhood; red line: gene fusions; dark-blue line: gene co-occurrence. Others: yellow line: text mining; black line: co-expression; baby-blue line: protein homology.

Our GWAS identified the SNP rs1561573 in 1p33, located in the intronic region of the *TRABD2B* gene. The principal analysis of protein–protein interaction networks (PPIs), represented by colored nodes in Figure 5, showed an association between the *TRABD2B* and aminoadipate aminotransferase (*AADAT* or *KYAT 2*) genes, indicating an analogous expression pattern across many RNAseq datasets [23]. The PPI analysis also showed an association between *TRABD2B* and kynurenine aminotransferase 1 (*KYAT1*) and kynurenine aminotransferase 3 (*KYAT3*) genes (Figure 5). Interestingly, the enrichment analysis showed a significant association (FDR = 0.0052) of *KYAT 1* and *3* with the Kyoto Encyclopedia of Genes and Genomes (KEGG) pathway: Selenocompound metabolism (hsa00450).

**Figure 6:**
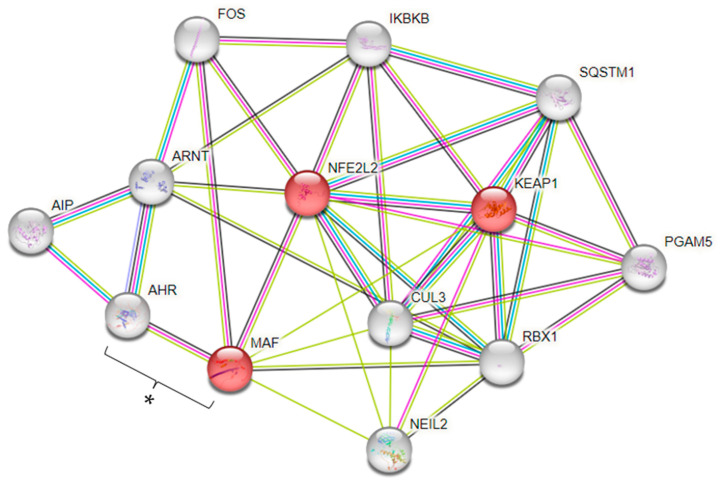
Protein–protein association network, using String (PPI enrichment *p*-value: 1.58 × 10^−5^). Network nodes represent proteins. Red nodes indicate genes involved in the *NRF2-ARE* regulation (WP4357). Edges represent protein–protein associations. Known Interactions—light-blue line: from curated databases; pink line: experimentally determined. Predicted Interactions—green line: gene neighborhood; red line: gene fusions; dark-blue line: gene co-occurrence. Others: yellow line: text mining; black line: co-expression; baby-blue line: protein homology. * Indicates the involvement between *AHR* and *MAF* genes.

Our GWAS also identified the *MAF* SNP rs425664. The WikiPathways enrichment analysis of *MAF* indicated its connection with Kelch-like ECH-associated protein 1 (*KEAP1*) and Nuclear Factor Erythroid 2-related factor 2 (*NFE2L2*), which are involved in the nuclear factor erythroid 2 p45-related factor 2-antioxidant-responsive elements (*NRF2-ARE*) regulation (WP4357) (FDR = 7.70 × 10^−5^) (Figure 6). Also, the PPI analysis indicated an association between *MAF* and Aryl hydrocarbon receptor (*AHR*) genes (Figure 6).

We also found biological interactions between Se deficiency and the SNP rs10444656 in the *SPATA13* gene, associated with thyroid metabolism, and the SNP rs6592284 in the *HIKESHI* gene, associated with body homeostasis.

## 4. Discussion

In the present study, an intriguing characteristic regarding the study population was serum Se deficiency despite adequate Se intake, a unique condition that has not yet been explored in the literature. This highlights the importance of studying an individual’s intrinsic factors, such as population variability and genetic variations, that could contribute to Se deficiency and help understand the biological mechanisms behind this condition [4].

In admixture populations, it is essential to explore whether candidate SNPs previously identified as associated with complex disorders in non-admixed populations also display association signals [33]. Since previous GWASs of Se concentrations were based on European populations, estimating the impact of population structure could avoid misinterpretations in other populations [7,9,11,14]. In the present study, the admixed Brazilians had a large proportion of European ancestry (71%), corroborating with the study of Kaibara et al. (2021) [33]. The authors replicated association signals on eight candidate regions previously found in European populations, indicating the possibility of transferability of polygenic risk scores from European studies to admixed Brazilian populations [33].

The present study identified 12 SNPs associated with serum Se deficiency in individuals from an admixture Brazilian population (*p*-value < 5.0 × 10^−4^). One of the most promising findings of our study was the SNP rs1561573 in the *TRABD2B* gene. *TRABD2B*, also known as *TIKI2*, is a metalloprotease involved in several processes, including negative regulation of the Wnt signaling pathway, positive regulation of protein oxidation, and positive regulation of protein-containing complex assembly [34]. The current literature did not describe the straight connection between TRABD2B and selenometabolism. Nonetheless, the Wnt pathway could converge between *TRABD2B* and selenometabolism since both interact with this metabolic pathway differently [35,36,37,38].

The Wnt pathway is a highly conserved system that regulates complex biological processes, playing crucial roles in embryogenesis, homeostasis, and regeneration [39,40]. Disruption of Wnt signaling activity results in a spectrum of abnormalities and diseases, including cancer, metabolic disease, and degeneration [39,40]. Many factors regulate the canonical Wnt signaling pathway. *TRABD2B* is a well-known regulator of Wnt/β-catenin signaling that targets Wnt proteins to affect signal transduction [40]. The Wnt signaling could also be modulated by Se concentration, selenoproteins, and Se species, reinforcing the importance of Se speciation studies [24,35,36,37]. Previous studies demonstrated that Se deficiency, SELENOP loss, and selenomethionine activate the Wnt signaling pathway [35,36,37]. On the other side, inhibition of the pathway due to increased degradation of β-catenin was observed in cell lines in the presence of 1,4-phenylene bis(methylene)selenocyanate, methylseleninic acid, and sodium selenite [36]. Further, an intriguing colocalization of GPX2 and the Wnt system was observed in the intestinal tract [36].

The PPI analysis indicated interactions between *TRABD2B*, *AADAT*/*KYAT 2*, *KYAT 1*, and *KYAT 3*. Although TRABD2B interactions with *KYAT 1* and *KYAT 3* were determined by text mining, in PPI analysis, all interactions constitute the larger superset of ‘functional protein–protein associations’ or ‘functional protein linkages’ [23]. Thus, the SNP rs1561573 in the TRABD2B gene could affect the functions of all related genes found in the protein–protein association network.

*KYAT 1* is a multifunctional enzyme that plays a significant role in Se-methyl selenocysteine (MSC) metabolism [23,41]. MSC is an organic methylated Se compound that has drawn attention because of its high bioavailability and favorable pharmacokinetics [42]. Also, the metabolites formed by MSC enzymatic transformation have been reported to possess pronounced tumor-specific cytotoxic activity by altering numerous cell signaling pathways [42]. *KYAT 1* utilizes MSC as a substrate to produce methylselenol (MS), pyruvate, and ammonium via β-elimination and β-methylselenopyruvate (MSP) via transamination [41,42]. *KYAT 3* also has a cysteine S-conjugate beta-lyase activity and can transaminate L-selenomethionine [41].

Unlike the other two kynurenine aminotransferases, *AADAT*/*KYAT 2* does not actively participate in Se metabolism but appears to be critical for the thyroid hormone (TH) metabolism since it catalyzes the transamination of T4 and T3 to TK4 and TK3, respectively [43]. This result is exciting since Se is critical for the function of the thyroid, and it is particularly more abundant in this gland than in most other organs [44].

We also found the SNPs rs425664 and rs10444656 in *MAF* and *SPATA13* genes, respectively. *MAF* and *SPATA13* were previously identified as susceptibility loci for thyroid stimulating hormone (TSH) levels, thyroid cancer risk, and thyroid function [43,45,46,47]. Thus, polymorphisms in these genes were strongly associated with impaired thyroid metabolism [43,45,46,47].

As mentioned above, Se is an essential element for thyroid function [43]. On the other hand, Mitagg et al., 2010 demonstrated the role of TH in regulating selenoprotein expression and Se status [48]. Using murine model systems, they indicated that TH positively regulates serum Se and SELENOP levels and implies a self-amplifying cycle of decreasing TH levels, causing reduced Se availability, which in turn impairs the activation of T4 by the Se-dependent deiodinases and the Se-dependent function of the hypothalamus–pituitary–thyroid axis [48]. These findings empowered our results in which SNPs in genes related to thyroid function (*AADAT/KYAT 2*, *MAF*, and *SPATA13*) could contribute to serum Se deficiency.

Apart from thyroid function, our analysis also indicated the involvement of *MAF* in *NRF2-ARE* regulation. *NRF2* is a transcription factor that orchestrates the adaptive response to an oxidative challenge by transcriptional upregulation of antioxidant and detoxifying phase 2 enzymes [36]. Remarkably, experimental studies demonstrated not only that Se regulated the *NRF2* pathway but Se status was affected in an *NRF2*-dependent manner, suggesting that impaired *NRF2* expression requires adequate Se supplementation to maintain normal antioxidant functions by synthesizing selenoproteins to protect the body against damage from oxidative stress [36,49,50].

Another interaction found in PPI analysis was between *MAF* and the aryl-hydrocarbon receptor (*AHR*). *AHR* is a member of transcription factors involved in the metabolism of drugs, lipids, and circadian rhythm [50]. Studies have demonstrated the connection between Se homeostasis and *AHR*, especially in environmental pollutant toxicity models [51,52,53].

The Gene Ontology Analysis indicated the involvement of *HIKESHI* in cellular response to stress (GO:0033554) and cellular response to heat (GO:0034605). *HIKESHI* is a nuclear import carrier for HSP70 that operates during heat shock stress [54]. *HIKESHI* mediates the nucleoporin-dependent translocation of ATP-bound HSP70 proteins into the nucleus, attenuating and reversing heat shock-induced nuclear phenotypes [54]. This result may bring an essential warning since the recent threats of climate change and global warming are predicted to increase the Earth’s temperature in the coming decades and, thereby, heat stress (HS) to the organisms [55]. The HS is suggested to increase free radical production and lower the concentrations of serum trace minerals involved in antioxidant defense in the body, such as Se [55]. HS also increases body metabolism, leading to increased nutrient requirements, which are attributed to HS-induced increased excretion of nutrients [55]. Recently, the murine study by Aderao et al., 2023 found that under prolonged HS conditions, the dietary Se requirement may be increased to 460 ppb for improving the antioxidant status and humoral immune response, and cytokine levels, modulating the thyroid and insulin hormone, and the selenoproteins mRNA expression of rats [54]. These results could support the hypothesis that polymorphisms in the *HIKESHI* gene could affect its capacity to counteract HS stress damage functions in the human body, influencing dietary Se requirements under HS conditions.

The present study has several limitations. First, the sample size was small for the GWAS of Se deficiency. More studies, including larger samples, are necessary to confirm the associations of identified variants with Se deficiency. However, to our knowledge, this is the first GWAS of Se deficiency in an admixed population that presents an unusual characteristic: Se deficiency despite sufficient Se intake. Our findings are significant in the context of increasing interest in the relationship between Se deficiency and metabolic pathways indirectly related to Se regulation. Here, we highlighted the influence of SNPs in genes that participate in thyroid hormone metabolism, NRF2-ARE, and Wnt pathways, as well as heat stress in the Se deficiency. Second, none of the SNPs reached the conventional genome-wide significance threshold (*p* < 5 × 10^−8^). Over the last decade, a p-value threshold of 5 × 10^−8^ has been considered in GWAS to classify significant associations and control false-positive associations [30,32]. However, studies have revised this strict threshold, especially considering substantial experimental and methodological advances in biostatistics/bioinformatics and to account for the lower allele frequency spectrum used in many recent GWASs [30,31,32]. A lower p-value threshold can also be observed in previous GWASs of Se concentrations in which potentially relevant SNPs were identified [7,9,11].

## 5. Conclusions

In conclusion, this is the first GWAS reporting the association between Se deficiency and SNPs in genes indirectly related to Se regulation in an admixed population that presents an unusual characteristic: Se deficiency despite sufficient Se intake. Our results reinforce the complexity of the Se interactions in the human body. Nevertheless, despite the promising results, it remains unclear how the genetic variation in these genes affects Se concentrations. Therefore, further studies are required to address the role of the SNPs in *TRABD2B, MAF, SPATA13*, and *HIKESHI* and the consequences to the Se metabolism.

## Figures and Tables

**Table 1:** Serum selenium deficiency-related single nucleotide polymorphisms.

SNP	Chr: Position	Gene	HWE * (*p*-Value)	MAF (%)	Allele	Mixed-Model ^#^ (*p*-Value)	Max-Stat (*p*-Value)
rs1561573	1:48227943	*TRABD2B*	0.1183	48.8	C/T	6.60 × 10^−5^	3.08 × 10^−4^
rs806792	6:26198916	*H3C4*	0.0892	10.2	A/G	1.72 × 10^−4^	4.57 × 10^−5^
rs9383382	6:10067722	*OFCC1*	0.3704	22.1	T/C	7.56 × 10^−6^	6.25 × 10^−5^
rs4478892	10:55634794	*PCDH15*	0.9051	31.1	G/A	1.18 × 10^−4^	3.72 × 10^−4^
rs6592284	11:86055901	*HIKESHI*	0.4897	18.3	T/C	3.42 × 10^−4^	2.35 × 10^−5^
rs2201161	12:99631219	*ANKS1B*	0.2116	45.1	A/G	6.11 × 10^−6^	1.70 × 10^−6^
rs10444656	13:24671601	*SPATA13*	0.6674	39.6	T/C	1.27 × 10^−4^	1.31 × 10^−4^
rs1241636	14:25354440	*STXBP6*	1.0000	44.4	C/T	2.26 × 10^−4^	1.95 × 10^−4^
rs10483637	14:55157430	*SAMD4A*	0.5579	15.6	C/T	1.01 × 10^−4^	6.67 × 10^−5^
rs8017018	14:79505543	*NRXN3*	1.0000	45.6	T/C	8.53 × 10^−5^	7.11 × 10^−5^
rs425664	16:79282227	*MAF*	0.6023	44.7	A/C	1.67 × 10^−4^	8.04 × 10^−5^
rs6509701	19:53384185	*ZNF320*	0.4966	34.9	C/T	4.27 × 10^−5^	2.11 × 10^−5^

Note: * Exact Tests of HWE proposed by Wigginton, Cutler, and Abecasis (2005). ^#^ Genome-wide mixed-model association analysis of serum selenium deficiency with sex, BMI, and age as covariates. *ANKS1B*: ankyrin repeat and sterile alpha motif domain containing 1B; Chr: Chromosome; *H3C4*: H3 clustered histone 4; HWE: Hardy–Weinberg Equilibrium; *HIKESHI*: heat shock protein nuclear import factor; *MAF*: MAF bZIP transcription factor; MAF: Minor Allele Frequency; Max-stat: maximum-statistical analysis; *NRXN3*: neurexin 3; *OFCC1*: orofacial cleft 1 candidate 1; *PCDH15*: protocadherin related 15; *SAMD4A*: sterile alpha motif domain containing 4A; SNP: single nucleotide polymorphism; *SPATA13*: spermatogenesis-associated 13; *STXBP6*: syntaxin binding protein 6; *TRABD2B*: TraB domain containing the 2B; *ZNF320*: Zinc Finger Protein 320.

## Data Availability

Data described in the manuscript, code book, and analytic code will be available upon request.

## Supplementary Materials

The following supporting information can be downloaded at https://www.mdpi.com/article/10.3390/nu16111627/s1, Supplementary Table S1: Common SNPs; Supplementary Table S2: Selenium and genotype association assuming the dominant model, adjusted by BMI, sex, and age; Supplementary Table S3: Selenium and genotype association assuming the recessive model, adjusted by BMI, sex, and age; Supplementary Table S4: Selenium and genotype association assuming the log-additive model, adjusted by BMI, sex, and age.
