# Supplementary material for: Genome-Wide Admixture and Association Study of Serum Selenium Deficiency to Identify Genetic Variants Indirectly Linked to Selenium Regulation in Brazilian Adults
Source: Nutrients. 2024 May 26;16(11):1627. doi: 10.3390/nu16111627 (PMC11175099; doi:10.3390/nu16111627)
Supplement: Supplementary file 1 [file nutrients-16-01627-s001.zip › Supplementary Table S1.pdf]

Supplementary Table S1. Common SNPs

SNP	SNP position	Gene	Gene symbol:Function	Gene location
rs10175198	chr2:45826975	protein kinase C epsilon	PRKCE : Intron Variant	2p21
rs10444656	chr13:24097462	spermatogenesis associated 13	SPATA13 : Intron Variant	13q12.12
rs10483637	chr14:54690712	sterile alpha motif domain containing 4A	SAMD4A : Intron Variant	14q22.2
rs10826846	chr10:30696152	supervillin family member 2, pseudogene	SVIL2P : Intron Variant	10p11.23
rs10839538	chr11:6277573	None		
rs10845611	chr12:12699448	G protein-coupled receptor 19	GPR19 : Intron Variant	12p13.1
rs1148191	chr10:28358210	None		
rs11652805	chr17:64991033	None		
rs118774	chr17:69964505	long intergenic non-protein coding RNA 1497	LINC01497 : Non Coding Transcript Variant	
rs12060889	chr1:91453501	None		
rs12331778	chr4:14398752	None		
rs1241636	chr14:24885234	syntaxin binding protein 6	STXBP6 : Intron Variant	14q12
rs12540120	chr7:42372808	None		
rs12593327	chr15:75661755	sorting nexin 33	SNX33 : 3 Prime UTR Variant	15q24.2
rs13114765	chr4:188469795	long intergenic non-protein coding RNA 1060	LINC01060 : Intron Variant	4q35.2
rs8017018	chr14:79039200	neurexin 3	NRXN3 : Intron Variant	14q24.3-q31.1
rs806792	chr6:26198688	H3 clustered histone 4; H2B clustered histone 7; H2A clustered histone 7	H3C4 : Intron Variant<br>H2BC7 : 2KB Upstream Variant<br>H2AC7 : 500B Downstream Variant	6p22.2
rs8180768	chr7:21617567	dynein axonemal heavy chain 11	DNAH11 : Intron Variant	7p15.3
rs9379577	chr6:23775367	None		
rs9382219	chr6:53600202	None		
rs9383382	chr6:10067489	orofacial cleft 1 candidate 1	OFCC1 : Intron Variant	6p24.3
rs9470848	chr6:38272309	BTB domain containing 9	BTBD9 : Intron Variant	6p21.2
rs9869315	chr3:175837516	None		
rs1398084	chr12:99476849	ankyrin repeat and sterile alpha motif domain containing 1B	ANKS1B : Intron Variant	12q23.1
rs1472254	chr3:27385734	solute carrier family 4 member 7	SLC4A7 : Intron Variant	3p24.1
rs1561573	chr1:47762271	TraB domain containing 2B	TRABD2B : Intron Variant	1p33
rs1623277	chr12:130007765	None		
rs16904932	chr8:133490079	ST3 beta-galactoside alpha-2,3-sialyltransferase 1	ST3GAL1 : Intron Variant	8q24.22
rs1863390	chr3:187619420	None		
rs2201161	chr12:99237441	ankyrin repeat and sterile alpha motif domain containing 1B	ANKS1B : Intron Variant	12q23.1

rs2583893	chr7:42705979	long intergenic non-protein coding RNA 1448	LINC01448 : Intron Variant	
rs369656	chr6:1214653	None		
rs3756325	chr5:140989274	protocadherin alpha 1	PCDHA1 : Intron Variant	5q31.3
rs3924999	chr8:32595840	neuregulin 1	NRG1 : Missense Variant	8p12
rs4082298	chr10:28357689	None		
rs425664	chr16:79248330	MAF bZIP transcription factor	MAF : Intron Variant	16q23.2
rs4478892	chr10:53875034	protocadherin related 15	PCDH15 : Intron Variant	10q21.1
rs4574840	chr8:56662896	None		
rs4784335	chr16:53975776	FTO alpha-ketoglutarate dependent dioxygenase	FTO : Intron Variant	16q12.2
rs6441805	chr3:43927304	None		
rs6509701	chr19:52880932	Zinc Finger Protein 320	ZNF320 : Synonymous Variant	19q13.41
rs6546856	chr2:73610624	None		
rs6592284	chr11:86344859	heat shock protein nuclear import factor hikeshi	HIKESHI : Non Coding Transcript Variant	11q14.2
rs7250095	chr19:318308	MIER family member 2	MIER2 : Intron Variant	19p13.3
