# Supplementary material for: Genome-Wide Admixture and Association Study of Serum Selenium Deficiency to Identify Genetic Variants Indirectly Linked to Selenium Regulation in Brazilian Adults
Source: Nutrients. 2024 May 26;16(11):1627. doi: 10.3390/nu16111627 (PMC11175099; doi:10.3390/nu16111627)
Supplement: Supplementary file 1 [file nutrients-16-01627-s001.zip › Supplementary Table S2.pdf]

Supplementary Table S2.: Selenium and genotype association assuming the dominant model, adjusted by BMI, sex and age

SNP	0 (%)	1 (%)	OR	lower	upper	p-value	AIC
**rs9383382**							
A/A	164 (67.5)	59 (45.4)	1.00	1.88	5.28	7.57E-06	377.1
A/G-G/G	79 (32.5)	71 (54.6)	3.16				
**rs1241636**							
A/A	92 (37.4)	24 (18.5)	1.00	1.60	5.13	0.000227	385.6
A/G-G/G	154 (62.6)	106 (81.5)	2.87				
**rs10483637**							
T/T	196 (78.7)	77 (58.3)	1.00	1.68	4.95	0.000101	387.0
T/C-C/C	53 (21.3)	55 (41.7)	2.88				
**rs8180768**							
T/T	156 (63.7)	107 (81.7)	1.00	0.19	0.62	0.00021	387.1
T/C-C/C	89 (36.3)	24 (18.3)	0.34				
**rs1398084**							
C/C	87 (35.4)	72 (55.8)	1.00	0.22	0.61	7.61E-05	382.6
T/C-T/T	159 (64.6)	57 (44.2)	0.37				
**rs4784335**							
A/A	80 (32.9)	74 (56.1)	1.00	0.20	0.56	1.86E-05	382.5
A/C-C/C	163 (67.1)	58 (43.9)	0.34				
