# Supplementary material for: Genome-Wide Admixture and Association Study of Serum Selenium Deficiency to Identify Genetic Variants Indirectly Linked to Selenium Regulation in Brazilian Adults
Source: Nutrients. 2024 May 26;16(11):1627. doi: 10.3390/nu16111627 (PMC11175099; doi:10.3390/nu16111627)
Supplement: Supplementary file 1 [file nutrients-16-01627-s001.zip › Supplementary Table S3.pdf]

Supplementary Table S3.: Selenium and genotype association assuming the recessive model, adjusted by BMI, sex and age

SNP	0 (%)	1 (%)	OR	lower	upper	p-value	AIC
**rs4478892**							
A/A-A/G	234 (94.7)	109 (82.6)	1.00	2.13	11.81	0.000118	388.1
G/G	13 (5.3)	23 (17.4)	5.02				
**rs2201161**							
T/T-T/C	211 (86.1)	83 (62.9)	1.00	2.1	6.89	6.11E-06	381.6
C/C	34 (13.9)	49 (37.1)	3.8				
**rs10175198**							
T/T-T/C	191 (78.6)	124 (94.7)	1.00	0.04	0.27	5.43E-08	370.9
C/C	52 (21.4)	7 (5.3)	0.10				
**rs10845611**							
C/C-T/C	194 (79.5)	126 (95.5)	1	0.08	0.55	0.000287	388.4
T/T	50 (20.5)	6 (4.5)	0.21				
**rs12593327**							
C/C-T/C	226 (91.9)	130 (100)	1.00	0	0	0.000345	385.7
T/T	20 (8.1)	0 (0.0)	0				
**rs1472254**							
G/G-A/G	193 (78.5)	125 (95.4)	1.00	0.08	0.51	0.00014	385.3
A/A	53 (21.5)	6 (4.6)	0.20				
**rs16904932**							
G/G-A/G	205 (83.0)	125 (96.9)	1.00	0.06	0.52	0.000264	384.3
A/A	42 (17.0)	4 (3.1)	0.17				
**rs3756325**							
G/G-A/G	208 (83.5)	126 (96.2)	1.00	0.07	0.55	0.000432	389.9
A/A	41 (16.5)	5 (3.8)	0.2				
**rs3924999**							
G/G-A/G	203 (82.9)	128 (97.0)	1.00	0.06	0.51	0.000231	388.4
A/A	42 (17.1)	4 (3.0)	0.17				
