# Supplementary material for: Genome-Wide Admixture and Association Study of Serum Selenium Deficiency to Identify Genetic Variants Indirectly Linked to Selenium Regulation in Brazilian Adults
Source: Nutrients. 2024 May 26;16(11):1627. doi: 10.3390/nu16111627 (PMC11175099; doi:10.3390/nu16111627)
Supplement: Supplementary file 1 [file nutrients-16-01627-s001.zip › Supplementary Table S4.pdf]

Supplementary Table S4.: Selenium and genotype association assuming the log-additive model, adjusted by BMI, sex and age

SNP	0 (%)	1 (%)	OR	lower	upper	p-value	AIC
**rs1561573**							
0,1,2	241 (65.1)	129 (34.9)	2.15	1.46	3.17	6.60E-05	381.7
**rs806792**							
0,1,2	246 (65.1)	132 (34.9)	2.76	1.59	4.79	0.000172	387.3
**rs6592284**							
0,1,2	248 (65.6)	130 (34.4)	2.29	1.45	3.61	0.000343	387.6
**rs10444656**							
0,1,2	245 (65.2)	131 (34.8)	2	1.39	2.88	0.000127	384.7
**rs425664**							
0,1,2	244 (65.2)	130 (34.8)	1.95	1.37	2.79	0.000167	385.1
**rs6509701**							
0,1,2	247 (65.5)	130 (34.5)	2.14	1.47	3.11	4.27E-05	383.6
**rs9470848**							
0,1,2	247 (65.5)	130 (34.5)	0.42	0.28	0.63	1.35E-05	381.4
**rs7250095**							
0,1,2	246 (65.6)	129 (34.4)	0.45	0.29	0.71	0.00031	383.9
